# Smoking aggravates ventricular arrhythmic events in non-ischemic dilated cardiomyopathy associated with a late gadolinium enhancement in cardiac MRI

**DOI:** 10.1038/s41598-018-34145-9

**Published:** 2018-10-23

**Authors:** Junbeom Park, Hye-Jeong Lee, Sook Kyoung Kim, Jeong-Eun Yi, Dong Geum Shin, Jung Myung Lee, Yookyung Kim, Young-Jin Kim, Boyoung Joung

**Affiliations:** 1 Department of Cardiology, College of Medicine, Ewha Womans University, Seoul, Korea; 2 Department of Radiology, Research Institute of Radiological Science, Yonsei University College of Medicine, Seoul, Korea; 3 Department of Biomedical Engineering, Medical College, Korea University, Seoul, Korea; 4 Division of Cardiology, Department of Internal Medicine, Gangneung Asan Hospital, Gangneung, Republic of Korea; 5 Department of Medicine, Graduate School, Kyung Hee University, Seoul, Korea; 6 Department of Radiology, College of Medicine, Ewha Womans University, Seoul, Korea; 7 Department of Cardiology, Internal medicine, Yonsei University Health System, Seoul, Korea

## Abstract

Smoking is known to increase cardiovascular events, but the association and mechanisms between smoking and ventricular arrhythmic events in dilated cardiomyopathy (DCMP) are unknown. The purpose of this study is to investigate the hypothesis that smoking is associated with sudden cardiac death (SCD) and ventricular arrhythmia in DCMP patients. We enrolled 378 patients who underwent cardiovascular magnetic resonance imaging (cMRI) and were diagnosed with DCMP at two general hospitals in Korea. The clinical data and left ventricular late-gadolinium enhancement (LV-LGE) of all patients were analyzed according to being never-smokers or smokers. Smokers were more likely to be male than never-smokers, but there was no other clinical difference between them. Smokers had a greater LV-LGE ratio, and multi-segment involvement of LV-LGEs. Smoking and a low left ventricular (LV) ejection fraction were significant predictors of the presence of LV-LGEs even after adjusting for optimal medical therapy. In addition, smokers had a higher fatal ventricular arrhythmic (FVA; sustained ventricular tachycardia, and ventricular fibrillation) and FVA + SCD, and ex-smokers had a similar FVA to never-smokers during 44.3 ± 36.4 months of follow-up. Finally, smoking independently increased the FVA + SCD even after adjusting for the clinical variables and LV-LGE. Smoking is associated with a multi-segmental involvement of LV-LGE and increased FVA + SCD in DCMP patients when compared to never-smokers.

## Introduction

Cigarette Smoking is a well-known risk factor for significantly increasing the cardiovascular morbidity and mortality^1^. In the U.S., approximately 140,000 premature deaths per year from cardiovascular disease are triggered by cigarette smoking^2^. Although the exact components and mechanism of cigarette smoking that are related to increased cardiovascular morbidity are unclear, cigarette smoking is known to increase inflammation, thrombosis, oxidation of low-density lipoprotein cholesterol, and trigger fibrotic processes in body tissues including atrial muscle^3–6^. Cardiac fibrosis has a strong correlation with systolic dysfunction, diastolic dysfunction, and abnormal electrical excitation; thus, it is also known to increase the incidence of arrhythmias, ventricular tachycardia, and sudden cardiac attacks. Studies reveal that the extracellular matrix in between cardiac myocytes slows the electrical conduction and the myofibroblasts produce ectopic electrical activity, which all-together eventually form micro-reentrant circuits within the endocardial tissue, leading to arrhythmogenesis^3^. Therefore, cardiac fibrosis is suggested as a fundamental factor for predicting the cardiovascular morbidity and mortality by modifying the mechanical, electrical, and vasomotor function^7^. In the clinical field, cardiac fibrosis can be evaluated through late-gadolinium enhancement (LGE) by cardiovascular magnetic resonance (CMR) imaging^8^. Recent studies have shown an excellent agreement of the histologic pattern of cardiac fibrosis with *in vivo* CMR imaging. Using LGE-CMR imaging, the results exposed that the likelihood of the developing arrhythmias in dilated cardiomyopathy (DCMP) is higher in people with midwall fibrosis than in those without fibrosis. Moreover, it has been reported that myocardial fibrosis assessed by LGE-cMRI is associated with cardiovascular mortality and sudden cardiac death (SCD) in patients with dilated cardiomyopathy (DCMP)^9^. In fact, it is known that as fibrosis measured by the LGE-CMR increases by 3%, the all-cause mortality increases by a mean of 55%^7^. Therefore, the aim of this study was to investigate the association between smoking and myocardial fibrosis assessed by LGE-CMR in DCMP patients and to determine whether the smoking is associated with incidence of arrhythmic events due to myocardial fibrosis including ventricular arrhythmias and sudden cardiac death (SCD). We limited our case-group to non-ischemic cardiomyopathy (NICM) patients to exclude ventricular fibrosis associated with coronary artery disease^10^. Further, to support our hypothesis, we divided our group into smokers and never-smokers. Then we compared the amount of cardiac fibrosis within the two groups and analyzed the relationship between left ventricular fibrosis using the LGE by CMR imaging and the incidence of arrhythmic events including ventricular arrhythmias, and SCD.

## Methods

### Study population and design

The study protocol was approved by the institutional review boards of two general hospitals. The study protocol was approved by the Institutional Review Board of Yonsei University Health System and Ewha Womans University Mokdong Hospital and conducted in accordance with the tenets of the Declaration of Helsinki (EUMC 2015-07-046-016). All patients provided written informed consent. A total of 378 patients who were diagnosed with non-ischemic DCMP and underwent CMR imaging between May 2003 and December 2015 at two tertiary hospitals in Korea were retrospectively enrolled in this study. All patients underwent a detailed history and clinical evaluation including a 12-lead electrocardiogram (ECG), 2-dimensional transthoracic echocardiography, contrast enhanced CMR, and coronary angiography or coronary computed tomography (CT). DCMP was defined in accordance with the World Health Organization/International Society and Federation of Cardiology’s guidelines^11^. Thus, studies that were included in this analysis met the following criteria. (1) Patients with a left ventricular ejection fraction (LVEF) of <50% without regional wall-motion abnormalities, (2) LV diastolic dimension of >55 mm, (3) symptoms or signs of heart failure according to the Framingham criteria, and (4) the absence of significant coronary artery disease (CAD) on angiography or 3-dimensional cardiac CT (to ensure exclusion of >50% obstructions of one or more coronary arteries) were included in this study^10,12^. Patients with significant valvular heart disease of more than moderate to severe, hypertrophic cardiomyopathy, restrictive cardiomyopathy, prior cardiac surgery, or structural heart disease were excluded.

### Echocardiography measurements

All patients underwent trans-thoracic echocardiography (TTE; Sonos 5500, Philips Medical System, Andover, MA, USA or Vivid 7, GE Vingmed Ultrasound, Horten, Norway). The LVEF, chamber size (left atrial volume index [LAVI], LA dimension, wall thickness of the LV, and LV mass index [LVMI]), transmitral flow velocity (E wave, A wave), and tissue Doppler images of the mitral annular septal area (peak diastolic velocity [Em], peak systolic velocity [S’]) were acquired according to the American Society of Echocardiography guidelines^13,14^.

### The definition of smoking, alcohol, and medication history

The information on the exposure of smoking and alcohol was acquired from patient questionnaires or medical records at the first visit to the hospital. (1) smokers were defined as having a smoking exposure if they used more than 100 cigarettes in their lifetime (ex-smoker + current smokers), (2) never-smokers were defined as those with a smoking exposure of less than 100 cigarettes over their lifetime, (3) ex-smokers were defined as those with smoking cessation from at least 1 year prior, and (4) current smokers were defined as those with a smoking exposure of more than 100 cigarettes, and that had smoked within the last year. The definition of alcohol intake was described as a frequency of ≥1–3 times per month. The medication history of the patients was analyzed based on the chart review of their use of the following drugs. Angiotensin converting enzyme inhibitors (ACEis) or angiotensin receptor blockers (ARBs), beta-blockers (BBs), diuretics, aldosterone antagonists, statins, digoxin, and amiodarone were included, and the use of each medication was defined for a prescription of more than at least 3 months regardless of the dose.

### CMR imaging

All CMR imaging studies were performed using a 1.5-T scanner (Intera Achieva; Philips Medical Systems, Best, the Netherlandsor Philips Healthcare, Andover, MA, USA) with a phased array cardiac coil. electrocardiogram (ECG)-gated cine imaging was performed using a balanced steady-state free precession sequence with the following parameters: TR/TE, 3.4/1.7 ms; flip angle, 50°; field of view, 360 × 360 mm; matrix, 256 × 256; slice thickness, 8 mm; 25 phases per cardiac cycle; average number of signals, 1; and short axis planes encompassing the entire LV without a slice gap. Delayed enhancement imaging was performed 10 minutes after an intravenous injection of gadolinium (0.2 mmol/kg; gadoterate dimeglumine; Dotarem, Geurbet) at 2 mL/sec. Image acquisition was synchronized with the ECG in the mid-diastolic phase in order to minimize motion artifact. An inversion time (TI) was individually optimized to nullify the signal of the normal myocardium using a dedicated TI-determining sequence^15^.

### Image analysis

All CMR images were transferred to a dedicated software program for analysis (CMR42, Circle Cardiovascular Imaging, Calgary, Alberta, Canada). The images were reviewed by three radiologists (H.J.L, Y.J.K., and YKK with 8, 11 and 25 years of experience in cardiac imaging, respectively) in consensus. Cine images were analyzed to determine the ventricular volume and ejection fraction using a semi-automatic segmentation in the software. The LGE was assessed using a modified 16-segment model of the LV^16^, and each segment model was rearranged to the anterior, interventricular septum, inferior, and lateral wall. In addition, the junction between the LV and right ventricle (RV) was described as a focal, hyperintensity area confined to the junction of the RV wall to the anterior and/or posterior interventricular septum, and multi-segment involvement was defined as involvement of more than 3 segments. An LGE quantification was performed. A semiautomatic segmentation of the endocardial and epicardial borders was performed in each short axis image in order to obtain the myocardial volume. The LGE volume was quantified using a full width at half maximum (FWHM) technique and the region of interest was drawn in the area of the maximum signal intensity of a visible LGE for the FWHM threshold. The myocardial and LGE volumes were determined as the sum of each area for each slice, multiplied by the slice thickness. The percentage of the LGE was calculated by dividing the LGE volume by the myocardial volume, with a quotient multiplied by 100. We used the median value as the cut-off of the LGE volume in the DCMP patients, and in this study, the median value of the LGE volume was 3.5%. We analyzed the patients after dividing the groups into those with an LGE extent of ≥3.5% and those with an LGE extent <3.5%. The CMR imaging was evaluated by three radiologists in each hospital for the location, and quantification of the LGE. As previously reported^17^, the inter-observer agreement between the first two readers was substantial (kappa value = 0.827, p < 0.005). The intra-observer variability of the LGE quantification for the CV and ICC were 12.5% and 0.99 (95% confidence interval [CI] 0.97–0.99), respectively^17^.

### Follow up

After the standardized data collection was completed, all case records were reviewed for the purposes of obtaining additional information. Follow-up data were obtained by a detailed review of all medical records of all patients. Patients had regular follow-ups with or without a standard 12-lead ECG in the outpatient clinic every 3 to 6 months, or when symptoms occurred. Patients with symptoms suggestive of arrhythmias underwent ambulatory Holter monitoring (24 hours) and/or event recording. Patients without any symptoms were evaluated every year with 24-hour Holter monitoring. The analysis of the ECG Holter recordings focused on ventricular tachycardia (VT), either non-sustained (NSVT) or sustained (SuVT). NSVT was defined as a tachycardia (≥100 beats/min) of a ventricular origin that self-terminated within 30 seconds, and SuVT was defined as that which lasted for more than 30 seconds. Ventricular fibrillation (VF) that was identified by the QRS complexes had a markedly different morphology, axis, and amplitude and no obvious P waves were observed and the rate was irregular and usually greater than 300 beats per minute.

Implantable cardioverter defibrillators (ICDs) or cardiac resynchronization therapy devices with a defibrillator (CRT-D) that were implanted for primary prevention were decided by the physician’s discretion and patient’s willingness. A routine ICD or interrogation (at 1 and 3 months; and every 3 months thereafter) and ECG recordings at the time of the symptoms were used to document the occurrence of spontaneous VT during the follow-up period in all patients. An appropriate ICD intervention was defined as a device shock or anti-tachycardia overdrive pacing delivered in response to a VT and documented in the stored intracardiac ECG data. All interrogations were identified and adjudicated by two electrophysiologists (D.G.S. and J.B.P); who were blinded to the clinical data, including the CMR analyses. The outcomes of this study were defined as: (1) major ventricular arrhythmias (MVAs) including NSVT, SuVT, and VF and (2) fatal ventricular arrhythmias (FVAs), including SuVT and VF. We described SCD as an unexpected natural death from a cardiac cause within 1 hour from the onset of symptoms, in a person without any prior condition that would appear fatal^18^. In addition, the cause and date of death were confirmed by information from the National Population Registry of the Korea National Statistical Office, together with a review of all available clinical records at the time of death^17^.

### Statistical analysis

Continuous variables are reported as the mean ± standard deviation (SD). Continuous variables were analyzed using independent t-tests. Normality was determined using the Kolmogorov-Smirnov goodness-of-fit test. Categorical variables were reported as numbers and percentages and were analyzed using Pearson’s chi-squared tests or Fisher’s exact tests, as appropriate. Cox regression and Kaplan-Meier analysis were performed for predictors of FVA(+SCD) and smoking or never smoking groups, respectively. A P-value ≤ 0.05 was considered statistically significant.

## Results

**Figure 1:**
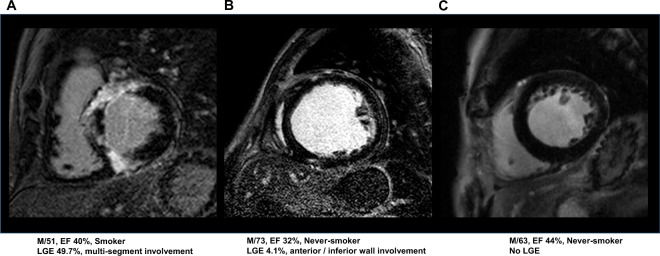
Typical example of an LV-LGE in a smoker (multi-segment involves) and never-smoker.

A total of 378 patients (62.2% male, 54.3 ± 14.4 years old) were enrolled for the analysis. Among them, there were 131 (34.7%) smokers, including 48 (12.7%) ex-smokers and 83 (22.0%) current smokers, and there were 247 (65.3%) never-smokers. Smokers more usually were male (95.4 vs. 44.5%, p < 0.001) and drank more alcohol (77.1 vs. 17.0%, p < 0.001) than never-smokers. However, there was no significant difference in the age and co-morbidities such as hypertension and diabetes (Table 1). Figure 1 shows the representative examples of an LV-LGE in a smoker and never-smoker in patients with DCMP. Regarding the cardiac function and MRI findings, the LVEF and end diastolic volume (EDV) also did not exhibit any significant differences between the two groups. However, smokers had a larger ratio of the LV-LGE than the never-smokers (9.3 ± 13.5% vs. 6.8 ± 11.2%, p = 0.059), and the proportion of patients with an LV-LGE of ≥3.5% (median values) was prominently higher in the smoking groups than never-smokers (58.8% vs. 45.3%, p = 0.019). In an analysis of the location of the LV-LGE, the smokers had a greater multi-segmental involvement (≥3 segments; 20.6% vs. 11.7%, p = 0.02) than the never-smokers (Table 2). In a subgroup analysis, the proportion of patients with an LV-LGE ≥3.5% was prominently higher in the ex-smoking groups than the current- and never smokers (69% vs. 53% vs. 46%, p = 0.014). In an analysis of the LV-LGE location, current- smokers had a greater multi-segmental involvement (23% vs. 11% vs. 16%, p = 0.038) than the never-smokers and ex-smokers (Supplement Table 1).

**Table 1:** Baseline characteristics.

	All (n = 378)	Never-smokers (n = 247)	Smokers (n = 131)	p value
Age (years)	54.3 ± 14.4	54.7 ± 15.1	53.3 ± 13.0	0.371
Male (n, %)	235 (62.2)	137 (44.5)	125 (95.4)	<**0**.**001**
Body surface area (m^2^)	1.7 ± 0.2	1.7 ± 0.2	1.8 ± 0.2	<**0**.**001**
Body mass index (kg/m^2^)	27.9 ± 35.9	26.4 ± 26.5	30.7 ± 48.1	0.337
Hypertension (n, %)	179 (47.4)	120 (48.6)	59 (45.0)	0.49
Diabetes (n, %)	113 (30.0)	75 (30.4)	38 (29.0)	0.766
Alcohol intake (n, %)	143 (37.8)	42 (17.0)	101 (77.1)	<**0**.**001**
NYHA class (n, %)	2.5 ± 0.8	2.5 ± 0.7	2.6 ± 0.8	0.194
Systolic BP (mmHg)	117.9 ± 18.4	117.9 ± 18.2	117.9 ± 18.8	0.981
Diastolic BP (mmHg)	74.6 ± 12.6	74.3 ± 12.2	75.2 ± 13.2	0.512
Presence of LBBB (n,%)	57 (15.1)	38 (15.4)	19 (14.5)	0.617
QRS duration (ms)	110.8 ± 27.7	109.8 ± 28.0	112.6 ± 27.1	0.355
Medication				
ACE inhibitor or ARB (n, %)	350 (92.6)	230 (93.2)	120 (91.6)	0.565
Beta blocker (n, %)	289 (76.5)	183 (74.2)	106 (80.9)	0.148
Diuretics (n, %)	311 (82.3)	206 (83.4)	105 (80.2)	0.417
Aldosterone antagonist (n, %)	249 (65.9)	160 (64.8)	89 (67.9)	0.563
Statin (n, %)	122 (32.3)	78 (31.6)	44 (33.6)	0.587
Digoxin (n, %)	138 (36.5)	87 (35.2)	51 (38.9)	0.464
Amiodarone (n, %)	12 (3.2)	8 (3.2)	4 (3.1)	0.867

p values < 0.05 are denoted by a bold font.

ACE = angiotensin-converting enzyme; ARB = angiotensin receptor blocker; BP = blood pressure; LBBB = left bundle branch block; NYHA = New York Heart Association.

**Table 2:** Cardiac MRI indices in never-smoking and smoking patients with DCMP.

	All (n = 378)	Never-smokers (n = 247)	Smokers (n = 131)	p
Geometric parameters				
LV ejection fraction (%)	26.3 ± 11.1	27.0 ± 11.6	24.9 ± 9.8	0.102
RV ejection fraction (%)	27.0 ± 21.0	28.0 ± 21.4	25.2 ± 20.3	0.281
LV EDV/BSA (ml/m^2^)	174.6 ± 97.7	166.5 ± 60.5	190.6 ± 141.6	0.157
RV EDV/BSA (ml/m^2^)	106.2 ± 42.9	101.8 ± 42.8	114.5 ± 42.1	0.063
The quantification of LV LGE				
Presence of an LV LGE (n, %)	276 (73.0)	173 (70.0)	103 (78.6)	0.086
The ratio of the LV LGE/LV (%)	7.6 ± 12.1	6.8 ± 11.2	9.3 ± 13.5	0.059
The ratio of the LV LGE ≥3.5% (n, %)	189 (50.0)	112 (45.3)	77 (58.8)	**0**.**019**
The location of LV LGE				
Anterior (n, %)	19 (5.0)	13 (5.3)	6 (4.6)	0.815
Interventricular septum (n, %)	173 (45.8)	108 (43.7)	65 (49.6)	0.328
Inferior (n, %)	28 (7.4)	18 (7.3)	10 (7.6)	0.848
Lateral (n, %)	14 (3.7)	9 (3.6)	5 (3.8)	0.894
Junction between LV-RV (n, %)	112 (29.6)	72 (29.1)	40 (30.5)	0.770
Multi-segment involvement (n, %)	56 (14.8)	29 (11.7)	27 (20.6)	**0**.**020**

p values < 0.05 are denoted by a bold font.

BSA = body surface area; EDV = end diastolic volume; LGE = late gadolinium enhancement; LV = left ventricle; RV = right ventricle; multi-segment involvement (≥3 segments).

LV fibrosis assessment by LV-LGE is a kind of LV remodeling in NICM patients with ventricular dysfunction. Just a low LVEF was associated with an increased LV-LGE (OR = 0.954, 95% CI 0.924–0.985, p = 0.004), but, the age, gender, and LV dilatation did not have a significant association with the formation of an LV-LGE in the multivariate analysis (Table 3). Especially, alcohol and smoking were associated with the presence of an LV-LGE, even after adjusting for the use of optimal heart failure medications. Finally, a low LVEF (OR = 0.953, 95% CI 0.920–0.987, p = 0.008), exposure to alcohol (OR = 3.239, 95% CI 1.196–8.774, p = 0.021), and smoking (OR = 3.694, 95% CI 1.396–9.775, p = 0.008) were associated with the formation of an LV-LGE (Table 3).

**Table 3:** The effect of smoking on the LV-LGE (Multivariate analysis).

	Adjusted for Clinical variables			Adjusted for Clinical variables + Medications		
	OR	95% CI	p value	OR	95% CI	p value
Age (per years)	1.008	0.984–1.032	0.521	1.003	0.978–1.029	0.81
Male	1.011	0.412–2.477	0.981	1.244	0.482–3.211	1.244
LVEDV/BSA (per 1 mL/m^2^)	0.999	0.996–1.003	0.728	0.999	0.996–1.002	0.999
LVEF (per 1%)	0.954	0.924–0.985	**0**.**004**	0.953	0.920–0.987	**0**.**008**
Hypertension	1.533	0.755–3.115	0.237	1.55	0.748–3.215	0.239
Diabetes	0.79	0.380–1.646	0.53	0.792	0.364–1.725	0.557
Alcohol	2.751	1.062–7.125	**0**.**037**	3.239	1.196–8.774	**0**.**021**
Smoking	3.124	1.211–8.061	**0**.**018**	3.694	1.396–9.775	**0**.**008**
Medication						
ACE inhibitor/ARB				0.837	0.230–3.052	0.788
Beta blocker				1.093	0.480–2.491	0.832
Loop diuretics				3.819	1.269–11.495	0.017
Aldosterone antagonist				0.664	0.255–1.725	0.4
Statin				0.881	0.430–1.803	0.728
Amiodarone				1.681	0.321–8.803	0.539

p values < 0.05 are denoted by a bold font.

ACE = angiotensin-converting enzyme; ARB = angiotensin receptor blocker; BSA = body surface area; LVEDV = left ventricular end diastolic volume; LVEF = left ventricular ejection fraction.

**Figure 2:**
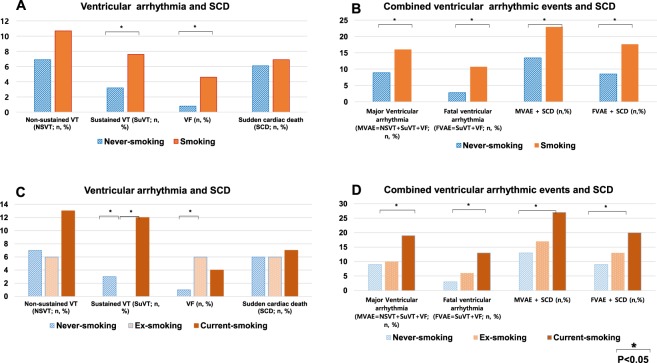
Ventricular arrhythmic events according to smoking in never-smokers and smokers with DCMP (**A**,**B**). Ventricular arrhythmic events according to smoking in the never-smoking, ex-smoking, and current-smoking patients with DCMP (**C**,**D**).

In an analysis of ventricular arrhythmias, the incidence of NSVTs did not exhibit any significant difference between never-smokers and smokers, but SuVTs (7.6% vs. 3.2%, p = 0.05) and VF (4.6% vs. 0.8%, p = 0.015) were significantly increased in smokers as compared to never-smokers (Fig. 2-A). When combined with ventricular arrhythmias and SCD, the incidence of MVAs (16% vs. 8.9%, p = 0.038), FVAs (10.7% vs. 2.8%, p = 0.002), MVA + SCDs (22.9%, vs. 13.4%, p = 0.018), and FVA + SCDs (17.6% vs. 8.5%, p = 0.009) were significantly increased in smokers as compared to never-smokers (Fig. 2-B). In a subgroup analysis, current smokers had the highest incidence of MVAs (10% vs. 9% vs. 19%, p = 0.030), FVAs (6% vs. 3% vs. 13%, p = 0.001), MVA + SCDs (17% vs. 13%. Vs. 27%, p = 0.016) and FVA + SCDs (13% vs. 9% vs. 20%, p = 0.010) as compared to ex- and never smokers, and ex-smokers had a relatively moderate risk compared to that of the other two groups (Fig. 2-D).

**Figure 3:**
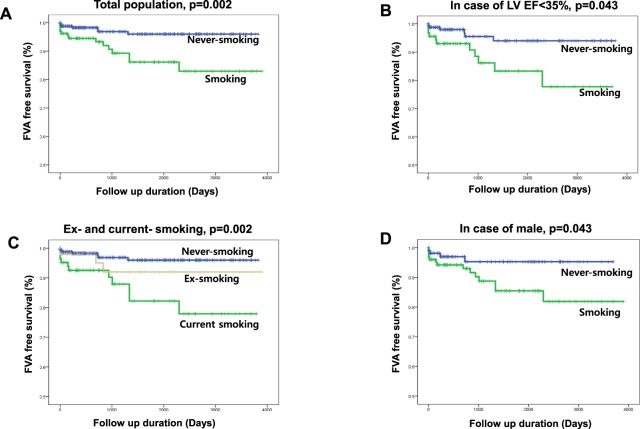
Kaplan-Meier curve of a fatal ventricular arrhythmia (FVA) according to smoking (**A**) in the total population (n = 378), (**B**) in patients with an LVEF <35% (n = 254), (**C**) in patients with a smoking history (ex-smokers and current smokers; n = 131), and (**D**) in male patients (n = 235).

**Figure 4:**
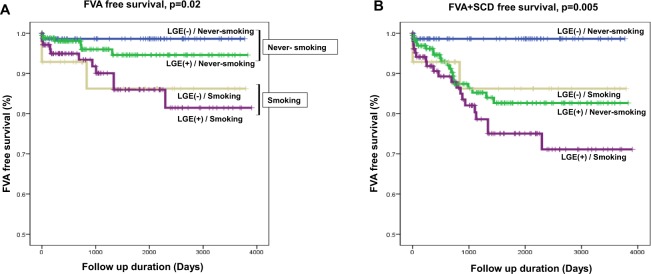
Kaplan-Meier curve of fatal ventricular arrhythmias (FVAs) and sudden cardiac death (SCD) according to an LGE and smoking. (**A**) FVA free survival, (**B**) FVA + SCD free survival.

During a follow-up duration of 44.3 ± 36.4 months, smokers had significantly increased FVA events as compared to never-smokers (Log Rank p = 0.002, Fig. 3-A), and this result also was consistent in the subgroup analysis of patients with a low EF (<35%) (Log Rank p = 0.043, Fig. 3-B). The FVA free survival of ex-smokers was located between never- and current smokers, which represented that stopping smoking may have protective effects on FVAs (Log Rank p = 0.002, Fig. 3-C). When considering most smokers were male (95.4%), male smokers also had significantly more FVA events than male never-smokers (Log Rank p = 0.043, Fig. 3-D). In an analysis combining an LGE and smoking, never-smokers without an LGE (−) had the lowest number of FVA events, and smokers with an LGE (+) had the highest number of FVA events. Especially smokers without an LGE (−) had a higher risk of FVAs than never-smokers with an LGE (+), and as a consequence, smoking had a greater association with the occurrence of FVAs than the presence of an LGE (Fig. 4-A). Also, in an analysis of FVA + SCD, an LGE (+) and smoking had consistent effects on ventricular arrhythmic events (Fig. 4-B). In a Cox regression multivariate analysis, a young age (HR = 0.96, 95% CI 0.95–0.996, p = 0.028), low body mass index (BMI) (HR = 0.83, 95% CI 0.716–0.962, p = 0.014), increased LVEDV (HR = 1.004, 95% CI 1.001–1.006, p = 0.004), alcohol intake (HR = 3.909, 95% CI 1.005–15.208, p = 0.049), and smoking (HR = 5.588, 95% CI 1.389–22.490, p = 0.015) were associated with FVAs or SCD. Finally, a low BMI and diastolic BP including smoking were common predictors of FVAs and FVA + SCD in patients with DCMP (Table 4), in addition, in the ROC curve analysis, the AUC area of the FVA group including smoking was 0.822, and the AUC area in the FVA + SCD group was 0.787, respectively (Supplement Fig. 1).

**Table 4:** Cox regression multivariate analysis of fatal ventricular arrhythmias (FVAs) and sudden cardiac death (SCD) in patients with DCMP.

Predictors	FVA				FVA + SCD			
	Univariate		Multivariate		Univariate		Multivariate	
	HR (95% CI)	P	HR (95% CI)	p	HR (95% CI)	p	HR (95% CI)	p
Age	0.968 (0.941–0.996)	0.026	0.96 (0.925–0.996)	**0.028**	0.988 (0.968–1.008)	0.228	0.975 (0.948–1.002)	0.072
Male	3.839 (1.130–13.039)	0.031	1.925 (0.395–9.386)	0.418	2.187 (1.080–4.428)	0.030	0.919 (0.302–2.795)	0.882
BMI (per 1 kg/m^2^)	0.870 (0.760–0.997)	0.046	0.83 (0.716–0.962)	**0.014**	0.901 (0.819–0.991)	0.032	0.866 (0.776–0.966)	**0.010**
LVEDV/BSA (per 1 ml/m^2^)	1.001 (0.998–1.003)	0.487	1.002 (0.998–1.005)	0.442	1.002 (1.000–1.003)	0.029	1.004 (1.001–1.006)	**0.004**
LVEF <35%	1.679 (0.491–5.740)	0.188	1.098 (0.210–5.749)	0.912	3.671 (1.131–11.914)	0.030	3.114 (0.574–16.890)	0.188
Presence of LGE	1.752 (0.589–5.213)	0.314	1.196 (0.287–4.986)	0.806	4.087 (1.461–11.429)	0.007	3.488 (0.899–13.539)	0.07
Hypertension	0.508 (0.205–1.260)	0.144	1.048 (0.322–3.409)	0.937	0.573 (0.310–1.060)	0.076	0.735 (0.299–1.811)	0.504
Diabetes	0.196 (0.046–0.844)	0.029	0.257 (0.052–1.279)	0.097	0.474 (0.228–0.987)	0.046	0.749 (0.287–1.951)	0.554
Systolic BP (per 1 mmHg)	0.982 (0.958–1.007)	0.163	0.998 (0.953–1.045)	0.936	0.976 (0.959–0.994)	0.008	1.014 (0.982–1.047)	0.401
Diastolic BP (per 1 mmHg)	0.961 (0.923–1.001)	0.058	0.922 (0.853–0.997)	**0.042**	0.942 (0.915–0.971)	0.000	0.913 (0.865–0.965)	**0.001**
Alcohol	1.200 (0.506–2.849)	0.679	3.909 (1.005–15.208)	**0.049**	1.209 (0.666–2.196)	0.533	1.887 (0.679–5.248)	0.224
**Smoking**	3.732 (1.506–9.247)	0.004	**5.588** (1.389–22.490)	**0.015**	2.043 (1.131–3.692)	0.018	**3.916** (1.259–12.173)	**0.018**

p values < 0.05 are denoted by a bold font. BP = blood pressure; BSA = body surface area; LGE = late gadolinium enhancement; LVEDV = left ventricular end diastolic volume; LVEF = left ventricular ejection fraction.

## Discussion

The findings of the present study can be summarized as follows: (1) In patients with DCMP, smokers were more likely to be male, and have multi-segmental involvement, (2) smoking and alcohol intake were associated with the presence of an LV-LGE, regardless of the optimal use of HF medications and a geometric change in the LV, (3) smokers had an increased number of FVAs as compared to the never-smokers, and stopping smoking within more than 1 year may have a protective effect on FVAs, and (4) smoking was an independent predictor of FVAs or FVA + SCD in patients with DCMP.

The LGE-CMR has been routinely used for the assessment of myocardial fibrosis in patients with DCMP^8^. The reason is that the extent of the myocardial fibrosis can be determined by the LGE^19^, the presence of which predicts cardiac events occurring in patients with DCMP^8^. In various cardiovascular diseases, the detection of fibrosis is a useful assessment of myocardial viability^20^, and the presence of fibrosis is a better marker of VT inducibility than the LVEF^21^. The available evidence also has proven that myocardial fibrosis, demonstrated by LGE-CMR, is an important predictor of cardiac mortality in non-ischemic cardiomyopathy^17,22^. Therefore, it has been reported that the LGE-CMR might be a useful tool for selecting suitable patients for primary ICD implantations in patients with non- ischemic cardiomyopathy^23^. In the present study, an increased LV-LGE was associated with a low LVEF in patients with DCMP. This is in agreement with the findings from the previous studies^8^. We also found that an LV-LGE in DCMP patients was associated with a worse prognosis such as SCD and FVA events. These results are based on the theories of the previous studies and suggest that an evaluation of LV-LGEs in patients with DCMP is clinically important.

It has been reported that smoking is associated with poorer outcomes in patients with DCMP^24,25^. Additionally, the association between smoking and cardiomyopathy has also been proposed by several animal studies^26,27^. Possible mechanisms underlying the association between smoking and non-ischemic cardiomyopathy include cardiac muscle damage by a disturbance in the oxidative processes and cardiac susceptibility after viral infections^27,28^. Although it is well known that smoking increases the cardiovascular mortality and morbidity, the association between smoking and an LV-LGE in patients with DCMP is not yet clear. In this study, we found that the smoking was associated with the presence of an LV-LGE, and smokers had a greater ratio of LV-LGEs than never-smokers. Especially, alcohol and smoking were associated with the presence of an LV-LGE, even after adjusting for the use of optimal heart failure medications. Additionally, in our data, the incidence of SuVTs and VF was significantly increased in smokers as compared to never-smokers. These findings suggest that smoking might be related to an increase in an LV-LGE formation and the incidence of FVAs in patients with DCMP.

Myocardial fibrosis is caused by a variety of factors, including alcohol intake, diabetes, hypertension, and smoking. Fibrosis due to alcohol intake is known to be caused by an increased diastolic stiffness and diastolic dysfunction of the left ventricle^29^. The mechanism of myocardial fibrosis due to hypertension has been reported to be caused by left ventricular diastolic dysfunction^30,31^. In addition, several cellular mechanisms of diabetic myocardial fibrosis have been reported to include an impaired excitation-contraction coupling, reduced coronary flow reserve, inefficient energy production, and fibrotic remodeling^32^. In previous reports, hypertension and diabetes influenced the formation of more myocardial fibrosis, so that, thus causing ventricular arrhythmias^33,34^. However, in our data (Table 1), there was no difference in the comorbid diseases between the two groups. Further, in Table 3, hypertension and diabetes had no association with the formation of an LGE (fibrosis). Although, no difference was found between the comorbid diseases between the groups, diabetes and hypertension singly may alter the myocardial vulnerability and cause arrhythmogenesis. The mechanisms of myocardial fibrosis in DCMP are complex and include inflammation, a genetic predisposition, micro-vascular ischemia, and neurohumoral changes^35^. Especially, smoking is one of the most important modifiable risk factors for various cardiovascular disease. Further, myocardial and endothelial cells are the targets for which tobacco smoke exerts its effects. Smoking has been reported to affect endothelial cell dysfunction such as endothelial cell injury, increased endothelial permeability, nitric oxide production, and the binding of inflammatory cells forming atherosclerotic plaques^36,37^. There was also a report that gene expressions related to metabolism, hypoxia, and a response to hormone stimulation were differentially expressed in smokers as compared to non-smokers^38^. Furthermore, one of the mechanism by which smoking affects cardiovascular disease is through chronic sympathetic activation as demonstrated by the increase in plasma epinephrine concentration levels, heart rate, systolic, diastolic, blood pressure and CO levels. Thus, these results suggest that smoking is a potential mechanism for inducing myocardial fibrosis and increasing the risk of ventricular arrhythmias^39,40^. Based on these mechanisms, our results showed that the pattern of fibrosis due to smoking was not exactly matched with the territory of the coronary arteries. Further, these kinds of patterns, such as multi-segment involvement including many conductive channels, could induce more ventricular arrhythmias. Yoshida A. *et al*. reported that a coexisting LV-LGE and perfusion-metabolism mismatch predict future cardiac events in patients with DCMP^41^. Moreover, multi-segmental involvement of fibrosis may represent many viable tissues and conductive channels for re-entrant circuits between fibrosis. Further, this finding is consistent with previous reports, which ventricular arrhythmias needed conductive channels for re-entrant circuits in the peri-scar region^42^. The location of the LV-LGE as well as the amount of an LV-LGE were also important in the prognosis of the patients. In our study, in an analysis of the location of the LV-LGE, smokers had a more multi-segmental involvement compared to never-smokers. Multi-segmental involvement of fibrosis was not matched with usual coronary territory, which may suggest a non-ischemic origin. Our study suggested that smoking and the LV-LGE location are clinically related and increase the risk of extended cardiomyopathy. Thus, we thought that further study of hypoxia and the perfusion-metabolism of the myocardium by smoking is needed. However, we clinically showed that a coexisting LV-LGE and smoking had the highest incidence of FVAs and SCD. Finally, although the presence of an LV-LGE could affect the cardiac mortality in DCMP patients, smoking was also a very important risk factor regardless of the presence of an LV-LGE.

### Clinical implications

Our results suggest that the association between smoking and an LGE is an important factor for ventricular arrhythmias. Patients with LV-LGEs, smoking and a low LVEF should be recognized as having a high-risk for fatal ventricular arrhythmias in DCMP. Therefore, based on the guidance recommendations, major precautions for FVAs and SCD in smokers with an LGE are needed. Another important result of our study was that current smokers had the highest incidence of FVAs and FVA + SCD compared to that of the ex- and never smokers. Importantly, the FVA free survival of ex-smokers was located between never- and current smokers, which represented that stopping smoking may have protective effects on FVAs.

### Study Limitations

This study had several limitations. First, NICM patients were classified by coronary angiography (CAG) and 3-dimensional cardiac CT assessment. At least in the early-stage of DCMP patients^10^, ischemic cardiomyopathy (ICM) was excluded. However, if NICM patients continuously smoke, it was not possible to exclude that ischemic events occurred during follow-up. Second, this study was a retrospective analysis, and included a select group of patients referred for treatment of cardiomyopathy. The data used in the study were acquired through a retrospective review of the patient’s charts. Thus, there was selection bias and limitations on the data acquisition. Third, only a few patients among those who met the indication for primary prevention received an ICD or CRT-D implantation. Therefore, fewer arrhythmic events in the patients might have been recorded due to suboptimal detection methods. Fourth, the patients did not undergo cardiac biopsy for the evaluation of the definite etiology and myocardial fibrosis. However, we excluded patients with features suggesting cardiomyopathies other than DCMP on CE-CMR. Fifth, diabetes and hypertension can affect myocardial fibrosis formation as well as smoking and can cause arrhythmias. However, there are limitations in comparing the single effects of smoking and alcohol consumption, diabetes, and hypertension, respectively. Especially, cardiomyopathic patients intaking alcohol are addicted to smoking, and patients with hypertension may have diabetes together. Therefore, it is difficult to prove the individual effect of each on myocardial fibrosis.

## Conclusion

Our study is the first to show a relationship between smoking and the formation of an LV-LGE in patients with DCMP. Smokers had a greater ratio of the LV-LGEs than never-smokers. Especially, alcohol and smoking were associated with the presence of an LV-LGE, even after adjusting for the use of optimal heart failure medications. Moreover, smokers had increased FVAs as compared to never-smokers. Importantly, the FVA free survival of ex-smokers was located between never- and current smokers, which represented that stopping smoking may have protective effects on FVAs. Therefore, a combined analysis of LV-LGEs and smoking is very important in DCMP patients and may be a useful marker for selecting patients with a high risk of FVAs and SCD.

## Electronic supplementary material

Supplementary Information
