# Supplementary material for: Smoking aggravates ventricular arrhythmic events in non-ischemic dilated cardiomyopathy associated with a late gadolinium enhancement in cardiac MRI
Source: Sci Rep. 2018 Oct 23;8:15609. doi: 10.1038/s41598-018-34145-9 (PMC6199322; doi:10.1038/s41598-018-34145-9)
Supplement: Supplementary file 1 — Supplementary Information [file 41598_2018_34145_MOESM1_ESM.pdf]

SUPPLEMENTARY MATERIAL to

"Smoking aggravates ventricular arrhythmic events in non-ischemic dilated cardiomyopathy associated with a late gadolinium enhancement in cardiac MRI"

Junbeom Park M.D., Ph.D.[1*], Hye-Jeong Lee M.D., Ph.D.[2*], Sook Kyoung Kim, Ph.D. [1, 3], Jeong-Eun Yi MD, PhD[1], Dong Geum Shin M.D.[4], Jung Myung Lee, MD.[5], Yookyung Kim M.D., Ph.D.[6],Young-Jin Kim M.D., Ph.D.[2]†, Boyoung Joung M.D.,Ph.D.[7]†

[1]Department of Cardiology, College of Medicine, Ewha Womans University, Seoul, Korea

[2]Department of Radiology, Research Institute of Radiological Science, Yonsei University College of Medicine, Seoul, Korea.

[3]Department of Biomedical Engineering, Medical College, Korea University, Seoul, Korea

[4]Division of Cardiology, Department of Internal Medicine, Gangneung Asan Hospital, Gangneung, Republic of Korea

[5]Department of Medicine, Graduate School, Kyung Hee University, Seoul, Korea

[6]Department of Radiology, College of Medicine, Ewha Womans University, Seoul, Korea

[7]Department of Cardiology, Internal medicine, Yonsei University Health System, Seoul, Korea.

[* These authors contributed equally to this work]

[†Joint senior authors]

Table S1. Cardiac MRI indices in never-smoking, ex-smoking, and current-smoking patients with DCMP

	Never-smokers (n=247)	Ex-smokers (n=48)	Current-smokers (n=83)	p value
**Age (years)**	54.8±15.1	57.5±12.0	50.9±13.0	**0.024**
**Male (n, %)**	45%	96%	95%	**<0.001**
**Geometric parameters**				
**LV ejection fraction (%)**	27.1±11.7	27.0±10.6	23.8±9.4	0.087
**RV ejection fraction (%)**	28.0±21.3	29.0±20.4	23.3±20.1	0.219
**LV EDV/BSA (ml/m^2)**	256.5±127.2	272.0±126.3	343.6±273.5	0.117
**RV EDV/BSA (ml/m2)**	101.8±42.8	98.4±31.6	122.0±44.6	**0.024**
**The quantification of LV LGE**				
**Presence of an LV LGE (n, %)**	70	85	74	0.116
**The ratio of the LV LGE / LV (%)**	6.8±11.2	9.6±13.9	9.1±13.4	0.16
**The ratio of LV the LGE ≥3.5% (n, %)**	46	69	53	**0.014**
**The location of LV LGE**				
**Anterior (n, %)**	5	4	5	0.966
**Interventricular septum (n, %)**	44	56	46	0.364
**Inferior (n, %)**	7	9	8	0.942
**Lateral (n, %)**	4	4	4	0.972
**Junction between LV-RV (n, %)**	29	40	25	0.207
**Multi-segment involvement (n, %)**	11	16	23	**0.038**

p values <0.05 are denoted by a bold font.

BSA = body surface area; EDV = end diastolic volume; LGE = late gadolinium enhancement; LV = left ventricle; RV = right ventricle; multi-segment involvement (≥3 segments)

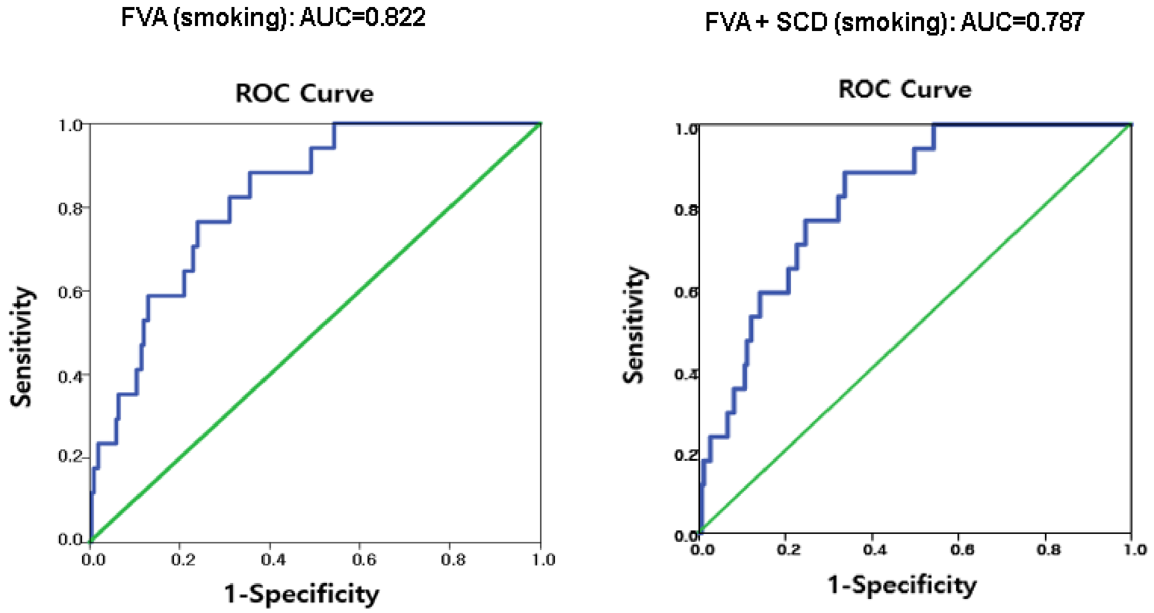

Figure S1: Receiver operating characteristic (ROC) curves for predicting the association of progression of ventricular arrhythmias with smoking.
